# Age-Related Decline in Vascular Responses to Phenylephrine Is Associated with Reduced Levels of HSP70

**DOI:** 10.3390/biom12081125

**Published:** 2022-08-16

**Authors:** Amanda A. de Oliveira, Valentina O. Mendoza, Fernanda Priviero, R. Clinton Webb, Kenia P. Nunes

**Affiliations:** 1 Laboratory of Vascular Biology, Department of Biomedical and Chemical Engineering and Sciences, Florida Institute of Technology, Melbourne, FL 32901, USA; 2 Department of Cell Biology and Anatomy, Cardiovascular Translational Research Center, University of South Carolina, Columbia, SC 29208, USA

**Keywords:** HSP70, middle-aged animals, vascular contraction, calcium, ROS

## Abstract

Aging impairs the expression of HSP70, an emergent player in vascular biology. However, it is unknown if age-related alterations in HSP70 are linked to a decline in arterial function. In this study, we test the hypothesis that the contributions of HSP70 to vascular contraction are diminished in middle-aged animals. We determined the basal levels of HSP70 in the aorta of young and middle-aged Sprague Dawley male rats using Western blotting. Functional studies were performed in a wire myograph system. Force development in response to phenylephrine was assessed in the presence or absence of extracellular calcium (Ca^2+^), and in aortic rings treated or non-treated with an HSP70 inhibitor. Fluorescent probes were used to evaluate vascular oxidative stress and nitric oxide levels. We report that middle-aged rats have significantly lower levels of HSP70. Blockade of HSP70 attenuated vascular phasic and tonic contraction in isolated aortas. It appears that a functional HSP70 is required for proper Ca^2+^ handling as inhibition of this protein led to reduced force–displacement in response to Ca^2+^ dynamics. Furthermore, middle-aged aortic rings exposed to the HSP70 inhibitor display higher reactive oxygen species levels without changes in nitric oxide. In summary, we show that middle-aged animals have lower levels of HSP70 in aortas, which associates with an age-related decline in vascular responses to α-1 adrenergic stimulation.

## 1. Introduction

Aging progressively associates with a decline in body function [1] and is a main non-modifiable risk factor for many conditions, including neurodegenerative and cardiovascular diseases (CVDs) [2]. It is estimated that by 2050, one in every six people will be 65 years old or more [3], which will likely increase the burden of age-related diseases overloading health systems worldwide. Vascular aging, which comprises at least structural, functional, and mechanical alterations in blood vessels, is a dynamic process encompassing the physiology of becoming older [4,5]. However, impaired contractile signaling in aged vascular structures leads to a reduction in arterial compliance, loss of arterial elasticity, and endothelial dysfunction [4]. The latter negatively affects endothelium-dependent vasodilation, compromising the functionality of vascular beds. This process is evidenced by decreased endothelial nitric oxide synthase (eNOS) expression, which diminishes NO availability, a condition that underlines various vascular complications [6]. Additionally, a continuous increase in reactive oxygen species (ROS) production during the aging process, triggering oxidative stress, harmfully impacts many signaling cascades with critical homeostatic functions [7].

Another important component of vascular aging, the decline in proteostasis (for review, please see [5]), could result from a reduction in the expression of molecular chaperones, including the 70 kDa, heat-shock protein 70 (HSP70). It is important to mention that the HSP70 family (a.k.a., HSPA) of proteins include 13 members in humans [8]. These proteins share high homology; consequently, a clear distinction between them is not always possible. Therefore, in this work, HSP70 is used as a generic term for the HSPA family. The levels of HSP70 (inducible isoform) may be a biomarker of biological age as adults and long-lived animals have higher levels of this protein compared with old and prematurely aged animals [9,10]. However, to the best of our knowledge, there is still limited data regarding vascular HSP70, especially in aged animals. α-1 adrenergic stimulation enhances the expression of HSP70 in isolated aortas, a process dependent upon the presence of Ca^2+^, and this activity is impaired in middle-aged animals [11]. While it is well-accepted that the induction of HSP70 has protective effects [12], experimental evidence in the vasculature, especially concerning vascular reactivity, is still lacking. This field gained an additional layer of importance as we recently showed that HSP70 is key for adequate vascular reactivity in young animals [13]. This novel function of HSP70 appears to be attributed to the fact that this protein affects Ca^2+^ handling mechanisms following adrenergic receptors’ stimulation in isolated aortas [14]. Still, it is unknown whether HSP70 plays a part in the aging-associated decline in vascular function, which could unveil new target(s) for this condition. In this sense, this study was designed to investigate the hypothesis that diminished HSP70 contributes to the aged-related decline in vascular responses to α-1 adrenergic stimulation in isolated aortas.

## 2. Materials and Methods

### 2.1. Animals

Young (12 weeks) and middle-aged (60 weeks) male Sprague Dawley rats were acquired from Taconic Biosciences and Envigo. Rats were housed in a controlled environment with unrestricted access to water and food, with light exposure cycles of 12 h. Body weight and glucose levels were assessed right before euthanizing the animals. The latter was evaluated in non-fasting rats with a commercially available glucose monitoring system. Animals were euthanized under anesthesia (isoflurane 5% in 100% O_2_) by exsanguination. The thoracic aorta was dissected and placed in cold physiological salt solution: (PSS in mmol/L: 130 NaCl, 4.7 KCl, 1.18 KH_2_PO_4_, 1.18 MgSO_4_ * 7H_2_O, 14.9 NaHCO_3_, 5.6 Dextrose, 1.56 CaCl_2_ * H_2_O, 0.026 EDTA). Subsequently, vessels were cleansed and sectioned into rings for histomorphometric analysis, Western blotting, functional studies, and detection of oxidative stress and nitric oxide.

### 2.2. Histomorphometric Analysis

Frozen thoracic aortas (2–3 mm) sections were cut on a cryostat (10 µm). Samples were immediately analyzed in a Zeiss Observer A1 AXIO inverted microscope with digital images collected using a Photometrics CoolSNAP MYO regular light camera under 20× magnification. Wall thickness was determined using the ImageJ software (NIH, Bethesda, MD, USA).

### 2.3. Western Blotting

Thoracic aortas were homogenized in Tissue Protein Extraction Reagent (ThermoFisher Scientific, n° 78510) supplemented with a Protease Inhibitor Cocktail (n° P8340). The BCA Protein Assay kit (ThermoFisher Scientific, n° 23225) was used to determine the total protein concentration in aortic preparations. Then, we loaded a total of 15 µg of protein into a 10% SDS-PAGE gel, which was transferred to a nitrocellulose membrane. Subsequently, membranes were exposed for 30 s to a Ponceau S solution, an image was acquired, and the membranes were washed for 5 min in Tris buffer with 1% Tween. To block non-specific binding sites, the membranes were incubated for 1 h at room temperature in a solution containing 5% non-fat dry milk diluted in Tris buffer with 1% Tween. A primary antibody against HSP70 (Cell Signaling, n° 4872, 1:1000), which was diluted in a mixture of Tris buffer with 1% Tween + 0.5 g of BSA, was used to probe the membranes overnight at 4 °C. This HSP70 antibody probes for the expression of three members of the HSP70 family; HSPA1A, HSPA1L, and HSPA8. The secondary anti-rabbit antibody (Cell Signaling, n° 7074, 1:10,000) was diluted in Tris buffer with 1% Tween. Then, the membranes were submerged with constant agitation into a solution containing the secondary antibody for 1 h at room temperature. To enhance the chemiluminescence, 1 mL of the SuperSignal West Femto Substrate (Thermo Fisher Scientific, n° 34095) was applied to each membrane. Then, immunoblots were revealed using the Chemidoc MP Imaging System (Bio-Rad, Hercules, CA, USA). The ImageJ software was used to quantify the density of the bands, which were normalized to the expression of GAPDH (Cell Signaling, #8884, 1:5000).

### 2.4. Functional Studies

We assessed vascular reactivity in aortic rings (2 mm) from young and middle-aged rats using a DMT620M multi-wire myograph system (Danish Myo Technology, Aarhus, Denmark). We previously investigated the contributions of HSP70 to the contraction mechanism of young animals [13,14,15]; therefore, in this work, we did not expose young aortic rings to the HSP70 inhibitor. Rings were mounted with a resting tension of 15 mN/mm in chambers filled with PSS and gassed with carbogen (95% CO_2_ and 5% O_2_, 37 °C). During the equilibration period (approximately 1 h), the PSS was changed every 15 min and the resting tension readjusted. After that, the viability of the rings was tested with a high KCl (120 mmol/L) solution. To prepare this solution, we modified the PSS to have the concentration of NaCl equimolar to KCl. Viable rings were washed and allowed to return to the resting tension (around 30 min).

#### 2.4.1. Time-Force Curves

Samples were incubated for 30 min with vehicle or VER155008 (10^−5^ mol/L; DMSO diluted), an HSP70 inhibitor [16]. VER155008 targets the product of the following genes of the HSP70 family: HSPA1A, HSPA5, and HSPA8. Therefore, we are unable to pinpoint the exact family member responsible for the inhibitor’s effects. Then, rings were stimulated with a single dose of phenylephrine (10^−5^ mol/L), and the force development was recorded for 10 min. The relationship between Time (T) and Force (F) was evaluated as previously described [14,15], with F being sampled every second. Briefly, we considered the moment we added phenylephrine to the chamber as $T_{0}$. After that, at any point in time, F was calculated by subtracting the F observed at $T_{0}$. The amplitude of the phasic ($A_{phasic}$) and tonic ($A_{tonic}$) parts of the curve were computed as follows. $A_{phasic}$ was calculated by subtracting the F observed at the moment the vessels shift from a rapid force development phenotype to a slow pattern, which was not consistent between animals, from the F observed at $T_{0}$. On the other hand, $A_{tonic}$ was determined by subtracting the F observed at $T_{600}$, which was the last point of the curve, from $A_{phasic}$ and the F observed at $T_{0}$.

#### 2.4.2. Ca^2+^ Protocol

We replaced the PSS with a Ca^2+^ free solution, supplemented with 1 mmol/L EGTA. After 3 min, we challenged the rings with phenylephrine (10^−5^ mol/L). We recorded the force developing for 10 min. Thereafter, we added Ca^2+^ to the solution to restore the initial PSS concentration of 1.56 mmol/L, and we evaluated the force generated for 15 min. Vehicle and VER155008 (10^−5^ mol/L) were added at $T_{0}$, which was the time in which we added the Ca^2+^ free solution to the chamber. The curves were calculated as previously described [14,15], with F being sampled every second. The E_max_ of the phasic ($E_{phasic}$) and tonic ($E_{tonic}$) parts of the curve, which represent force development in response to Ca^2+^ efflux from the sarcoplasmic reticulum (SR) and Ca^2+^ influx via plasmalemmal channels, respectively, were calculated as follows. The former was computed by subtracting the higher point of contraction after the addition of phenylephrine in Ca^2+^ free PSS from the measurement observed at $T_{0}$, and the latter by subtracting the F observed in the last point of the curve from the F observed at $T_{0}$. Curves were plotted showing that the samples were stable in the 5 min preceding the addition of the Ca^2+^ free PSS.

### 2.5. ROS and NO Detection

Thoracic aortas from both groups were cut into 2–3 mm rings. Rings were incubated with vehicle or VER155008 (10^−5^ mol/L) diluted in PSS for 6 h in a CO_2_ incubator at 37 °C. All samples were frozen in OCT compound at −20 °C. Transverse sections of the rings (10 μm) were obtained in a cryotome. Subsequently, in the dark, slides were incubated for 30 min with diaminofluorescein (DAF)-FM, dihydroethidium (DHE), and 2′,7′-dichlorodihydrofluorescein diacetate (H2DCFDA) (ThermoFisher Scientific, n° D23844, D11347, and D399, 10 μmol/L diluted in PBS, respectively). DPTA (100 μmol/L, n° D6518) was added to slides receiving the DHE probe. Images were acquired with equal exposure time for all samples on a Zeiss Observer A1 AXIO inverted microscope under 20× magnification. The Photometrics CoolSNAP MYO camera was used to acquire digital images. The ImageJ software was used to measure fluorescence in images without the background. Results are expressed as arbitrary units. We modified representative images for brightness and contrast using the same parameters.

### 2.6. Statistical Analysis

Data are depicted as means ± SEM. The sample size for the number of animals used for all sets of experiments is denoted as n. To determine significance between two and three groups, Student’s *t*-test and one-way ANOVA were used, respectively. The D’Agostino and Pearson test was used to confirm sample normality, and Pearson correlation (*p*) was computed when appropriated. A *p*-value ≤ 0.05 indicates statistical significance. All analyses were performed using the software GraphPad Prism, version 5.0.

## 3. Results

**Figure 1:**
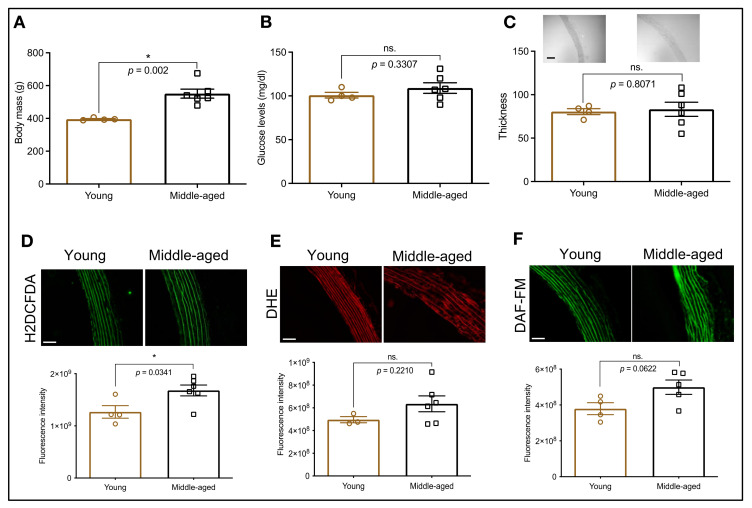
Animal profile. Body mass (**A**), glucose levels (**B**), and thoracic aorta thickness (µm); (**C**) in young and middle-aged animals. Levels of hydrogen peroxide (**D**), superoxide (**E**), and nitric oxide (**F**) were indirectly evaluated with fluorescent probes. The fluorescent intensity is displayed as arbitrary units. Scale bar: 50 µm. Data are expressed as mean ± SEM. (*n* = 4–6, except for the young group in panel E where *n* = 3), * *p* < 0.05 and ns. *p* > 0.05 vs. young.

To characterize the animals used in this study, we first determined the body weight and glucose levels of young and middle-aged rats. We found that, compared with young animals, middle-aged rats display increased body weight, but similar glucose levels (Figure 1A,B). The aging process is linked to arterial stiffening and the continuous production of ROS, triggering oxidative stress, which leads to abnormal signaling cascades, and ultimately, disruption of homeostatic functions. Therefore, we also assessed wall thickness, oxidative status (H2DCFDA and DHE), and nitric oxide availability (DAF). We observed that, compared with young animals, middle-aged rats have similar wall thickness (Figure 1C), but higher levels of ROS (Figure 1D; H2DCFDA). Conversely, when we used the DHE probe, we did not find augmented ROS levels in these animals (Figure 1E). Interestingly, middle-aged rats also appear to be leaning towards greater levels of nitric oxide (Figure 1F), suggestive of a compensatory mechanism.

### 3.1. HSP70 Expression in the Aorta: An Age-Dependent Process

**Figure 2:**
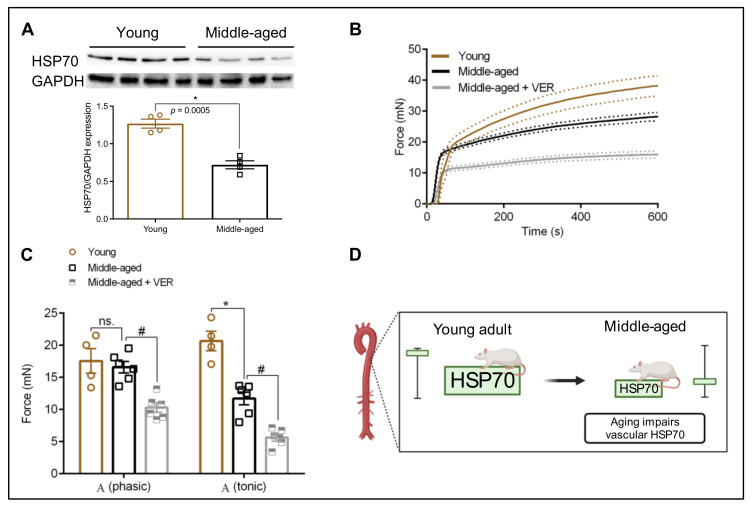
Inhibition of HSP70 further impairs vascular phasic ($A_{phasic}$) and tonic ($A_{tonic}$) contraction in the aorta of middle-aged rats. (**A**) Protein expression levels of HSP70 (HSPA1A, HSPA1L, and HSPA8) in aortas of young and middle-aged rats. Total protein expression levels were normalized to GAPDH. (**B**) Time vs. force curves for aortic samples isolated from young and middle-aged rats and stimulated with phenylephrine (10^−5^ mol/L) for 10 min. Samples from middle-aged animals (*n* = 6) were incubated with VER1550008 (10^−5^ mol/L) for 30 min before exposure to phenylephrine. The continuous line represents the mean, and the traced lines depict the SEM. (**C**) E_max_ of the phasic ($A_{phasic}$) and tonic ($A_{phasic}$) parts of the curve. (**D**) Schematic demonstrating that aging impairs vascular HSP70 expression. Panel D was created with BioRender.com (accessed on 16 July 2021). Data are expressed as mean ± SEM. (*n* = 4–6), * *p* < 0.05 and ns. *p* > 0.05 vs. young, and # *p* < 0.05 vs. middle-aged.

The aging process affects the expression levels of HSP70 in a tissue-specific manner [10]. HSP70 is a pervasive protein recently associated with assisting vascular contraction in young animals [13,14,15]. Here, we confirmed that, compared with young animals, middle-aged rats present a drastic reduction in levels of HSP70, as a measure of three HSP70 family members (HSPA1A, HSPA1L, and HSPA8) in the aorta (Figure 2A,D). Considering the emergent role of HSP70 in the vasculature and its pivotal functions in preventing increased oxidative stress, this remarkable difference supports the idea that decreased levels of HSP70 are likely detrimentally affecting vascular contractility.

### 3.2. Blockade of HSP70 Intensifies the Age-Related Decline in Tonic Contraction

We previously demonstrated that the blockade of HSP70 with a small molecule inhibitor, which targets this protein via its ATPase domain [16], and impairs vascular phasic ($A_{phasic}$) and tonic ($A_{tonic}$) contraction in young animals of both sexes [15]. However, it is still unknown whether HSP70 impacts these parameters in older animals, which undergo vascular adaptations [1]. Here, we found that middle-aged animals display reduced force development in the tonic ($A_{tonic}$) part of the curve, but these animals have preserved phasic ($A_{phasic}$) contraction (Figure 2B,C). The addition of the HSP70 inhibitor impaired force development in both contraction phases without altering the pattern of the curve (Figure 2B,C), suggesting that, in middle-aged animals, similar to what happens in young animals [13,14,15], this protein is also key for maintaining the vascular ability to elicit contraction in response to agonist stimulation. Strengthening this argument, we observed a strong correlation between the levels of HSP70 and F development in response to α1-adrenergic stimulation (*ρ* = 0.7121).

### 3.3. Inhibition of HSP70 Exacerbates the Aging-Related Decline in Ca^2+^ Dynamics Mediated Vascular Contraction

**Figure 3:**
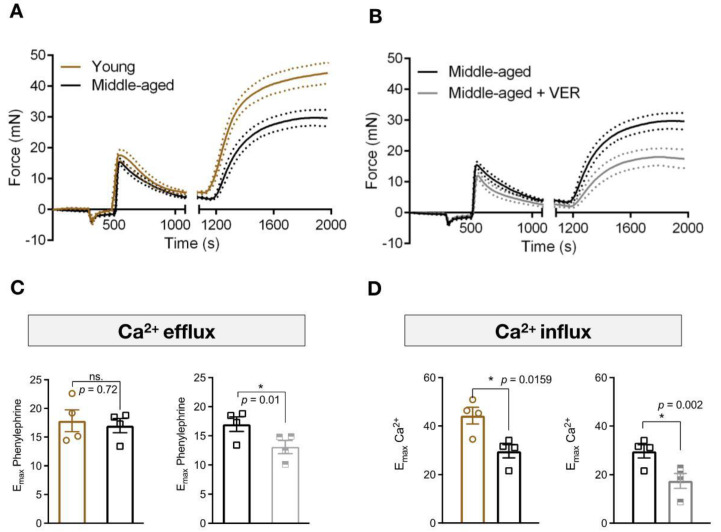
Blockade of HSP70 intensifies age-related decline in Ca^2+^ mediated contraction in aortas. (**A**,**B**) Aortic rings were stimulated with phenylephrine (10^−5^ mol/L) in Ca^2+^ free physiological salt solution (PSS), which was added 3 min before, for 10 min. Subsequently, the extracellular concentration of Ca^2+^ was restored and the force developed was analyzed for 15 min. Vehicle or VER155008 (10^−5^ mol/L) was added to the chamber at the moment the PSS was replaced by Ca^2+^ free PSS and were kept in the chambers for the entirety of the experiment (28 min). (**C**) E_max_ of phenylephrine in Ca^2+^ free PSS ($E_{phasic}$) and (**D**) E_max_ after the addition of Ca^2+^ ($E_{tonic}$). Data are expressed as mean (continuous line) ± SEM (traced lines). (*n* = 4), * *p* < 0.05 and ns. *p* > 0.05 vs. young or middle-aged.

There are significant alterations in the mechanisms involved in Ca^2+^ handling and sensitization in aged animals [17]. HSP70 is involved in muscle biology by affecting Ca^2+^ handling [14,18,19]. Therefore, we next conducted functional experiments in Ca^2+^ free PSS to observe the dynamics of force development in response to Ca^2+^ efflux from the SR and Ca^2+^ influx via plasmalemmal channels. In agreement with our previous set of experiments, we found that middle-aged animals have preserved force development in the phasic part of the contraction curve ($E_{phasic}$), but contraction is reduced in response to Ca^2+^ influx (Figure 3). More importantly, when we blocked the chaperone molecule HSP70, the ability of the preparations to elicit contraction was reduced in both parts of the curve (Figure 3). This indicates that, in the absence of this protein, rings display Ca^2+^ mishandling, resulting in a lower degree of contraction.

### 3.4. HSP70 and Vascular Oxidative Stress in Middle-Aged Animals

**Figure 4:**
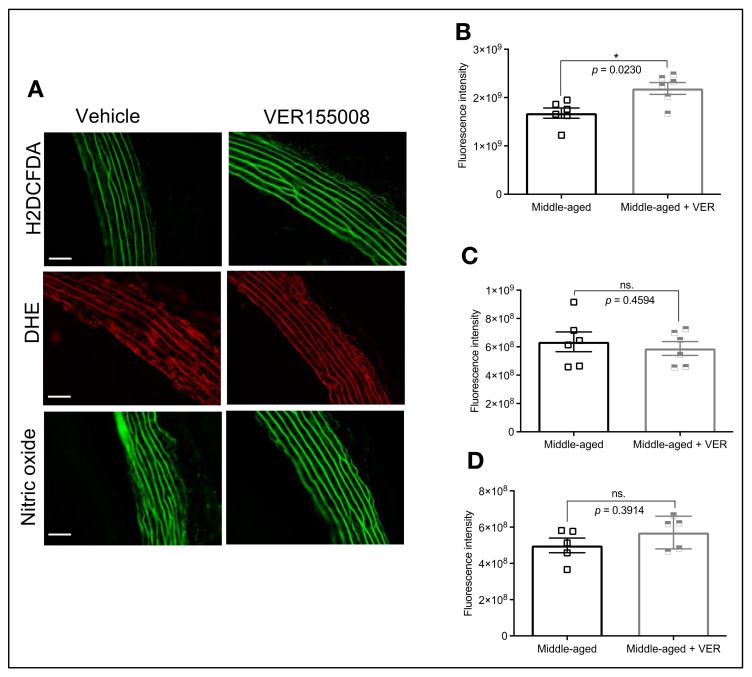
HSP70 Inhibition increases ROS production in the aorta of middle-aged animals but does not affect the levels of nitric oxide. (**A**) The fluorescent probes H2DCFDA, DHE, and DAF-FM were used to measure the levels of vascular oxidative stress and nitric oxide in isolated aortic rings from middle-aged rats incubated with vehicle or VER155008 (10^−5^ mol/L) for 6 h. Fluorescent intensity for H2DCFDA (**B**), DHE (**C**), and DAF-FM (**D**) are displayed as arbitrary units. Scale bar: 50 µm. Data are expressed as mean ± SEM. (*n* = 5–6), * *p* < 0.05 and ns. *p* > 0.05 vs. middle-aged.

The oxidative status of arterial structures is critical for vascular functionality. There is a link between HSP70 and ROS, but in our previous studies we reported that, under the conditions evaluated, the acute blockade of HSP70 does not lead to increased oxidative stress [13,15]. Since we found that middle-aged animals have reduced HSP70 and higher levels of ROS than young animals, we next sought to investigate if inhibiting this protein could exacerbate ROS production in isolated aortas. Under these conditions, the blockade of HSP70 augmented hydrogen peroxide production (Figure 4A,B), but not superoxide (Figure 4A,C) in middle-aged vessels. It is well accepted that superoxide reacts with nitric oxide to form peroxynitrite, which hampers nitric oxide availability [20]. In this case, we subsequently measured the levels of nitric oxide and, considering that the HSP70 did not affect superoxide, one would expect that the levels of this gaseous transmitter should be unaffected. Confirming our assumptions, we observed similar levels of nitric oxide between middle-aged rings exposed to vehicle and the HSP70 inhibitor (Figure 4A,D).

## 4. Discussion

In this study, we investigated the role of HSP70 in the vasculature of middle-aged rats (60 weeks), which roughly translates to 40 human years [21]. This is important since the risk of developing cardiovascular disease progressively increases as a person gets older [22]. In the United States, the incidence of cardiovascular diseases is already at approximately 40% in men and women aged 40 to 59 years [23]. The induction of HSP70 is a cellular defense mechanism associated with longevity [10]. Unfortunately, aged animals have a decline in their HSP70 induction capacity in various tissues [9]; and, there is a lack of information regarding this protein’s basal expression levels in isolated aortas of middle-aged animals. Furthermore, while we and others demonstrated a link between the levels of HSP70 and vascular contraction in young animals [13,14,15,24,25], it is still elusive whether reduced levels of arterial HSP70 can play a causative role in the age-related decline in vascular function, which could help explain why middle-aged animals display changes in force development following agonist stimulation. Here, we first show that aortas isolated from middle-aged animals have a reduction in the basal expression levels of HSP70 (Figure 2A), similar to what happens in other post-mitotic tissues [9] and in agreement with the observation that aged isolated aortas have lower HSP70 induction capacity [11]. Then, we provided initial evidence that impaired HSP70 could be a complex mechanistic insight into the pathways leading to age-related decline in vascular contraction, creating opportunities for future studies investigating this protein in the vascular biology of aged animals.

HSP70 exerts a key, yet emergent, role during receptor-mediated contraction in young animals [13,14]. In fact, the blockade of this protein impairs the amplitude of the fast ($A_{phasic}$) and slow ($A_{tonic}$) components of the contraction curve. Here, we expand these findings to show that a similar response also occurs in middle-aged animals (Figure 2B,C), and HSP70 basal levels are correlated with the magnitude of arterial contraction (Figure 2D). It is important to highlight that middle-aged animals preserved vascular phasic contraction ($A_{phasic}$) and impaired tonic ($A_{tonic}$) response (Figure 2B,C). If reduced HSP70 levels were the only factor contributing to force development in this condition, it is be reasonable to assume that both parts of the curve would have to be affected, which was not observed in our study. While it was demonstrated that aged animals present preserved responses to adrenergic stimulation in isolated aortas, there are significant variations in the mechanisms involved in Ca^2+^ handling and sensitization in comparison with young animals [17], suggesting that, as we age, physiological adaptations happen to preserve vascular function, and ultimately, extend lifespan. For example, aged skeletal muscle-fed arteries have reduced contraction following adrenergic stimulation, which occurs independently of the fact that they also display increased activation of Rho-kinase [26], a primary Ca^2+^ sensitizer [27,28]. In this sense, in middle-aged animals, alterations in HSP70 basal expression might impact Ca^2+^ handling mechanisms in both phases of the curve but Ca^2+^ sensitization might compensate for the downregulation in Ca^2+^ levels.

To understand how the blockade of HSP70 led to an impairment in force generation in response to phenylephrine, we next evaluated the impact of HSP70 inhibition in Ca^2+^ free PSS. Again, we observed that, compared with young animals, middle-aged rats display preserved force development in response to the emptying of the SR via IP3R (Figure 3A,B). However, there is a significant impairment in vascular tonic contraction in response to Ca^2+^ entry via plasmalemmal channels (Figure 3A,C). As we mentioned above, physiological alterations accompany the vascular aging process, and they are directly linked to calcium dynamics and sensitization [17]. It was demonstrated that, compared with young arteries, aged cerebral arteries (80–88 weeks) display identical maximal Ca^2+^ responses following stimulation of the IP3R, indicating that the aging process does not impact this process, despite the fact that these vessels present changes in the expression pattern of IP3R genes [29]. In this work, we found that the HSP70 inhibitor impaired the $E_{phasic}$ (Ca^2+^ efflux) and $E_{tonic}$ (Ca^2+^ influx) responses in middle-aged animals (Figure 3). We previously showed that HSP70 is essential for phasic and tonic force development in samples challenged with phenylephrine by interacting with IP3R-induced phasic contraction pathways and voltage-independent-mediated tonic force–displacement [14]. The decrease in force development in response to Ca^2+^ influx in aged arteries might occur as these vessels have lower expression of L-type Ca^2+^ channels (Cav1.2) [29,30], which results in reduced inward currents [30], as well as decreased Orai1 expression and SOCE-mediated contraction [31]. A previous study demonstrated that this pattern of decreased L-type Ca^2+^ channel expression is observed in aged aortas in physiological and pathological conditions [32]. Consequently, when we blocked HSP70, it seems that the major Ca^2+^ entry mechanisms were affected (i.e., voltage-dependent and voltage-independent channels), which resulted in a greater degree of impairment of force development. It is also essential to consider that the emptying of the SR is key for stimulating store operated Ca^2+^ channels and, since the inhibition of HSP70 led to a diminished $E_{phasic}$, it is possible that less Ca^2+^ leaving the SR led to a lower rate of Ca^2+^ entry via these channels ($E_{tonic}$). Either way, our results point to HSP70 as a central regulatory mechanism that could be targeted to improve vascular Ca^2+^ dynamics in middle-aged vessels.

Oxidative stress is another critical pathological mechanism associated with the development of vascular dysfunction [20] and Ca^2+^ dynamics during aging [33]. In this study, we observed that, compared with young animals, middle-aged rats display augmented ROS levels (Figure 1D,E). Additionally, we found that inhibition of HSP70 in middle-aged aortic rings led to increased oxidative stress (Figure 4A,B; H2DCFDA), which could be another mechanism contributing to reduced force development since some ROS can favor relaxation in vascular structures [34,35]. While the fluorescent probe H2DCFDA is widely used as an indirect indicator of hydrogen peroxide, concerns were raised regarding its selectivity [36]. It is well accepted that HSP70 impacts ROS production, including hydrogen peroxide, as mice lacking the HSP70-1 gene have impaired activity of SOD1 and SOD2 [37], which are enzymes responsible for catalyzing the dismutation of superoxide [38]. Another interesting aspect is the fact that middle-aged rats tend towards higher levels of nitric oxide (Figure 1F), which could be an initial compensatory mechanism that will decline over time. This could be happening because, even though an interplay exists between ROS and nitric oxide, a direct connection occurs with superoxide [39], which appears unaffected (Figure 1E) under our experimental conditions. We also found that the acute inhibition of HSP70 could not affect this parameter (Figure 4A,D), similar to what we previously observed in young animals [14].

We recognize that our work has limitations. First, we only tested the contributions of HSP70 to the contraction of male middle-aged animals. There is a link between estrogen and HSP70 [12], and as we previously showed, young female animals have lower levels of HSP70 in aortas [15]. However, how the aging process affects the contributions of HSP70 to the dynamics of vascular reactivity is unknown, calling for further studies. Second, following the Three Rs—replace, reduce, refine—guiding ethical principles in animal research, our sample size for each experiment included 4–6 animals; however, given the low dispersion of our datapoints, we were still able to derive meaningful results. Third, we did not account for variations in body composition and insulin sensitivity as mechanisms modulating HSP70 expression. Obesity-induced insulin resistance is linked to reduced levels of HSP70 [40]; and therefore, one could argue that aging is not the only factor contributing to reduced HSP70 levels in middle-aged animals. Lastly, we did not rescue the phenotype by overexpressing the protein, which also calls for additional investigation.

In summary, we showed that middle-aged animals have lower levels of HSP70, which associates with an age-related decline in vascular function. It seems that the mechanism by which reduced levels of HSP70 affect the reactivity of isolated aortas involves changes in Ca^2+^ dynamics and potentially augmentation of hydrogen peroxide production. Considering that age is a significant risk factor for developing a series of diseases, including vascular diseases, we believe that our work adds a critical piece of information about HSP70, a protein already known to play a critical role in age and age-related disorders. From a broader perspective, our findings corroborate the notion that the restoration of HSP70 would positively influence the vascular function of middle-aged animals. While further studies are warranted to limit this idea, our work strengthens the hypothesis that the levels of this protein are a suitable biomarker for vascular function and dysfunction.

## Data Availability

The original contributions presented in the study are included in the article, further inquiries can be directed to the corresponding authors.
